# Dammarane Sapogenins Improving Simulated Weightlessness-Induced Depressive-Like Behaviors and Cognitive Dysfunction in Rats

**DOI:** 10.3389/fpsyt.2021.638328

**Published:** 2021-03-26

**Authors:** Qiong Wang, Li Dong, Mengdi Wang, Shanguang Chen, Shanshan Li, Yongbing Chen, Wenlu He, Hong Zhang, Yongliang Zhang, Alberto Carlos Pires Dias, Sijin Yang, Xinmin Liu

**Affiliations:** ^1^Affiliated (T.C.M) Hospital, Sino-Portugal Traditional Chinese Medicine (TCM) International Cooperation Center, Southwest Medical University, Luzhou, China; ^2^Institute of Food Science and Technology, Chinese Academy of Agricultural Sciences (CAAS), Beijing, China; ^3^Department of Pharmacology, School of Pharmacy, Southwest Medical University, Luzhou, China; ^4^National Key Laboratory of Human Factors Engineering, China Astronaut Research and Training Center, Beijing, China; ^5^Department of General Surgery, Beijing Shijitan Hospital, Capital Medical University, Beijing, China; ^6^Department of Biology, University of Mihno, Braga, Portugal; ^7^Research Center for Pharmacology and Toxicology, Institute of Medicinal Plant Development (IMPLAD), Chinese Academy of Medical Sciences and Peking Union Medical College, Beijing, China

**Keywords:** Dammarane sapogenins, simulated weightlessness, depression, cognitive dysfunction, hindlimb suspension and isolation, rats

## Abstract

**Background:** Our studies demonstrated that the space environment has an impact on the brain function of astronauts. Numerous ground-based microgravity and social isolation showed that the space environment can induce brain function damages in humans and animals. Dammarane sapogenins (DS), an active fraction from oriental ginseng, possesses neuropsychic protective effects and has been shown to improve depression and memory. This study aimed to explore the effects and mechanisms of DS in attenuating depressive-like behaviors and cognitive deficiency induced by simulated weightlessness and isolation [hindlimb suspension and isolation (HLSI)] in rats.

**Methods:** Male rats were orally administered with two different doses of DS (37.5, 75 mg/kg) for 14 days, and huperzine-A (1 mg/kg) served as positive control. Rats were subjected to HLSI for 14 days except the control group during drug administration. The depressive-like behaviors were then evaluated by the open-field test, the novel object recognition test, and the forced swimming test. The spatial memory and working memory were evaluated by the Morris water maze (MWM) test, and the related mechanism was further explored by analyzing the activity of choline acetyltransferase (ChAT), acetylcholinesterase (AChE), and superoxide dismutase (SOD) in the hippocampus of rats.

**Results:** The results showed that DS treatment significantly reversed the HLSI-induced depressive-like behaviors in the open-field test, the novel object recognition test, and the forced swimming test and improved the HLSI-induced cognitive impairment in the MWM test. Furthermore, after DS treatment, the ChAT and SOD activities of HLSI rats were increased while AChE activity was significantly suppressed.

**Conclusions:** These findings clearly demonstrated that DS might exert a significant neuropsychic protective effect induced by spaceflight environment, driven in part by the modulation of cholinergic system and anti-oxidation in the hippocampus.

## Introduction

In long-term space missions, astronauts are exposed to complex and extreme environmental conditions (microgravity, circadian dys-synchrony, isolation, and confinement) which can negatively affect their physiological and psychological state and performance (1). Several studies have confirmed that the microgravity environment is an underlying factor for cognitive disorder. Cognitive impairment caused by weightlessness has also been reported earlier. For example, participants' reaction time of working memory is significantly decreased in head-down bed rest (HDBR) (2). Pavy-Le et al. (3) demonstrated that the balance between functional input and response output is disrupted, and HDBR detrimentally influence cognitive function under prolonged exposure to increased cephalic fluid distribution under simulated weightlessness (4). A human study of simulated weightlessness with 15° head-down tilt and 45° head-up tilt profoundly affects the brain function and mental arithmetic with impairment of memory processes (5). Additionally, simulated weightlessness increased cognitive impairment in rats (6). Effective countermeasures need to be explored regarding the health and performance of astronauts both during in- and post-flight conditions.

There is substantial evidence that Chinese herbs may provide a potential treatment of cognitive impairment induced by microgravity. *Panax ginseng* has been widely used as a tonic for over 2,000 years in China. It contains a diverse group of saponins known as ginsenosides with significant effects on cognition and depression. Dammarane sapogenins (DS), a kind of extracts derived from the *P. ginseng*, contains two main ingredients: 20(s)-protopanaxatriol (PPT) and 20(s)-protopanaxadiol (PPD) with neuroprotective effect (7, 8). In addition, DS caused improvement in cognitive impairment induced by sleep interruption in mice in a dose-dependent manner (9) and also has antidepressant-like properties (10). Head-down bed rest in human and hindlimb suspension (HLS) are popularly used in animals to simulate microgravity on earth. Liu et al. (2) reported that healthy young men (*n* = 16) who participated in the two-back task exhibited significantly lower reaction time, suggesting that the prolonged HDBR may have a detrimental effect on working memory. HLS has been recognized as a cost-effective ground-based rodent model mimicking weightlessness in microgravity research (11), which is acceptable by the National Aeronautics and Space Administration.

In the present study, the effect of DS on improving depressive-like behaviors and cognitive deficits induced by simulated weightlessness and isolation was addressed, as potential candidate for counteracting depression and memory deficiency induced in space environment.

## Methods

### Drugs and Reagents

**Figure 1:**
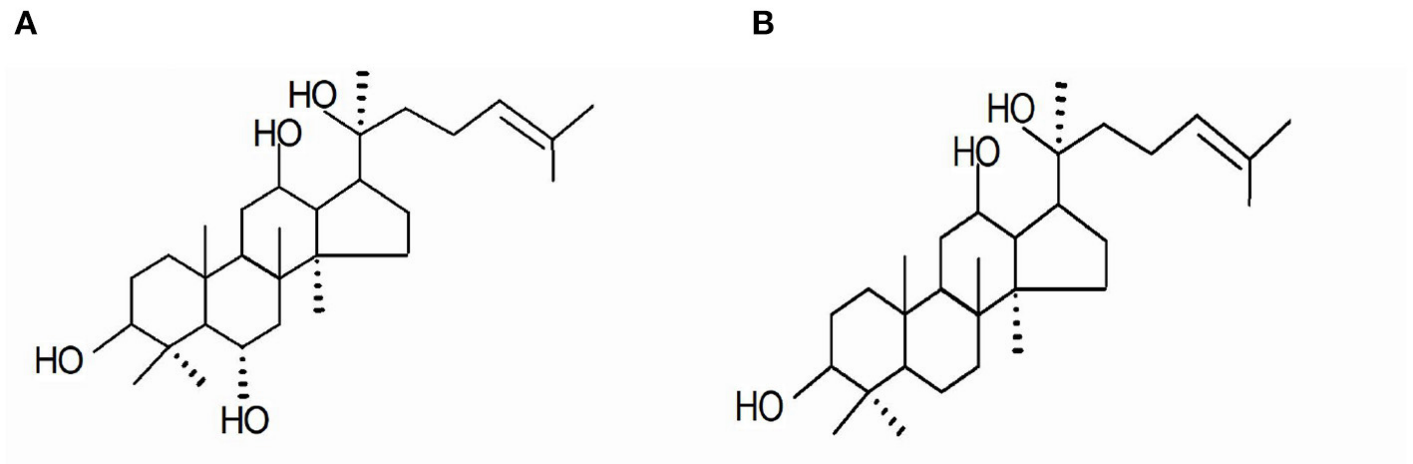
Main components residing in DS include the following: **(A)** 20(S)-protopanaxatrriol (PPT) and **(B)** 20(S)-protopanxadiol (PPD).

The DS was provided by Panagin Pharmaceuticals Incorporated (Canada, US patent 6888014B2, May 3, 2005, Huang D and Qi DF). It was prepared by alkaline hydrolysis of total ginsenosides derived from the stem and leaf of *Panax ginseng* C. A. Mayer and identified as described in our previous study. In brief, it contained 33% PPT and 16% PPD on the anhydrous basis (12) (Figure 1). Huperzine-A tablets were purchased from Shanghai Fudan Forward Pharmaceutical Co. Ltd. The acetylcholinesterase (AChE), choline acetyltransferase (ChAT), and superoxide dismutase (SOD) kits were purchased from Nanjing Jiancheng Bioengineering Research Institute (Nanjing, China).

### Apparatus

The computer-aided open-field test, the novel object recognition test, the computer-aided forced swimming test, and the computer-aided Morris water maze apparatus were developed jointly by the China Astronaut Research and Training Center and the Institute of Medicinal Plant Development, Chinese Academy of Medical Sciences.

### Animals

Fifty SPF male Sprague-Dawley rats (weighing 180–200 g) were purchased from Beijing Hua Fukang Biological Polytron Technologies Inc. (Beijing, China). They were housed individually in specific pathogen-free animal house with free access to standard diet and sterilized drinking water. Rats were maintained in a room with temperature of 22–25°C, relative humidity of 50–65%, and a 12-h light-dark cycle (lights on at 08:00 AM). Prior to experiments, rats were acclimatized for 3 consecutive days. All experiments were conducted according to the “Principles of Laboratory Animal Care” (NIH publication No. 86-23, 1996) and P.R. China legislation for the use and care of laboratory animals. The protocols were approved by the committee for the Care and Use of Laboratory Animals of IMPLAD, CAMS & PUMC, China (No. 2016515).

### Experimental Design and Treatment

The rats were randomly divided into five groups with 10 rats per group: control, hindlimb suspension and isolation (HLSI), HLSI + huperzine-A 1 mg/kg (positive control), HLSI + DS 37.5 mg/kg, and HLSI + DS 75 mg/kg, with the latter two serving as DS treated groups. Rats of the control group were housed as five per cage, while rats with the HLSI groups were kept alone per cage. All rats were housed in the same environment with a natural light-dark cycle. The open-field test, the novel object recognition test, the forced swimming test, and the Morris water maze test were performed after 14 days of HLSI.

DS and huperzine-A were dissolved in distilled water and orally administered at 10 ml/kg to rats in the HLSI + DS group and HLSI + huperzine-A group. The control animals and the HLSI group received distilled water (10 ml/kg). Drugs and water were administered daily to rats. Behavioral test was performed 1 h after drugs or water administration in rats.

### Hindlimb Suspension and Isolation Procedures

**Figure 2:**
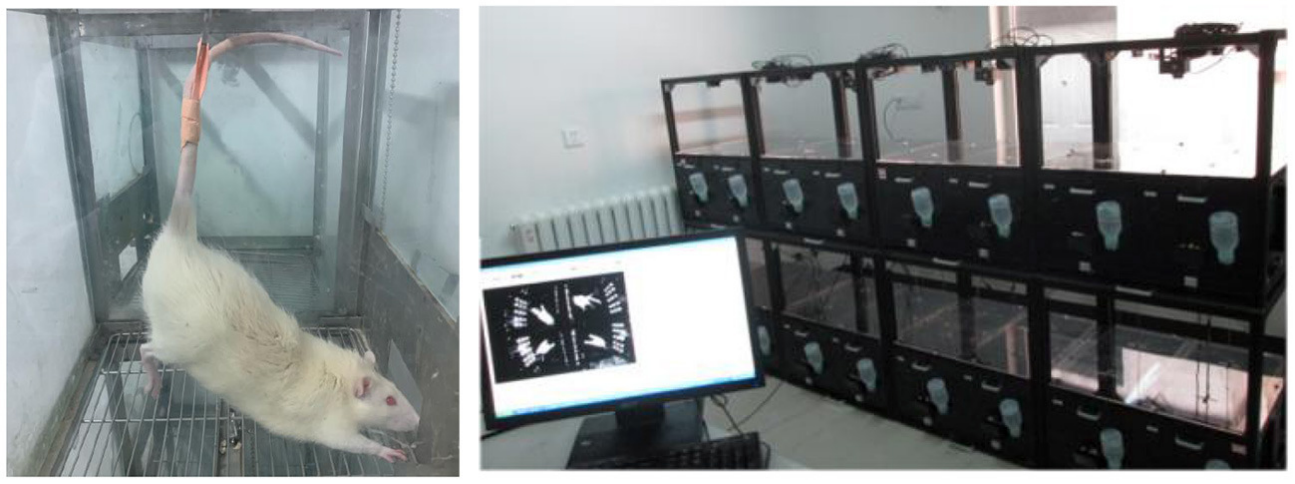
Rats in HLSI and the instrument for HLSI.

The hindlimb suspension is a classical method of simulating weightlessness. In order to reduce spontaneous activity and the damage to tail, a simulated weightlessness apparatus was developed (Chinese patent No. 201310228949.2) (Figure 2). In brief, rats were placed in a 26 × 26 × 30 cm black plexiglass box with animals isolated from each other. Rat tails were bound using medical adhesive tape and fixed with a small hook in a stainless chain mounted at the top of the cage, allowing free access to water and food. Rats were maintained at an angle of about 30° suspension between the floor and the body of the animal for 14 days, followed by behavior tests. After behavioral tests, rats were maintained HLSI again.

### The Open-Field Test

To assess the locomotor activity and the response to a novel environment, rats were placed into an open box (100 cm diameter, 50 cm height) with two 60 lx lighting on the ceiling and allowed to explore freely for 10 min. The computer-aided image analysis system for real-time detection test was employed during the test. After every experiment, the rats were returned to the cage and the open-field boxes were cleaned with 70% alcohol solution and dried. The arena was divided into two areas, the center area (central diameter 40 cm), and the peripheral area (left diameter). The total locomotor activity and the performance in the two arenas (with four paws) were evaluated. The parameters recorded included: total movement distance, average speed, duration of movement, movement distance in the central area, duration of movement in the central area, average speed in the central area, number of rearings during former 5 min (animal's double forelimbs leave the ground at the same time, or the two forelimbs are placed on the wall of the cage) (13).

### The Novel Object Recognition Test

After the open-field test, the novel object recognition test (NORT) was performed as described earlier (14). Firstly, the rats were placed in the box for 2 min for adaptation. After acclimatization period, a blue cylindrical object (5 cm × 5 cm in height and diameter) was introduced in the middle of the box. The time rats explored it for the first time was recorded for 10 min representing the exploration latency. The numbers of sniffing the object and the total time of sniffing were recorded as the numbers of exploring and the total time of exploration. The exploratory behavior is defined when the animals' mouth and nose are <1 cm away from the object or directly touching the object, but animals standing on objects are not considered explorations (15).

### The Forced Swimming Test

The force swimming test (FST) is a behavioral test used in rodents to assess anti-depressant-like behavior and performed as previously described (10). All experimental animals were first habituated to the testing room 30 min prior to testing. A pre-swimming (10 min) was conducted initially 24 h prior to the experiment. On the test day, all animals were subjected to the plexiglass cylinder (40 cm tall × 20 cm in diameter) filled with water (24°C) to a depth of 18 cm. Test sessions (5 min) were recorded by a video camera positioned directly above the cylinder. Animals were removed from their home cage and placed into the tank for a 5-min test. After drying, they were returned to their home cages. Water was replaced between animal experiments. Behaviors such as immobility, swimming, and climbing in the tank were conducted. Climbing was defined by the rat presenting its forepaws along the edge of the cylinder in an upward movement. Any horizontal movement was classified as swimming. Finally, immobility was defined as no additional movement required by the animal to maintain its head above water (16).

### The Morris Water Maze Test

This test was used to evaluate spatial learning ability that relies on distal cues to navigate from start locations around the pool to locate a submerged platform for escaping. The apparatus consisted of a circular pool (160 cm diameter, 50 cm height), filled with 22 ± 2°C water. In order to hide the platform, black ink was added to the water. In the pool, a platform (9 cm diameter) was placed at the center of a quadrant and adjusted 1.5 cm below the water level. Rats would start from two different starting points. It is worth mentioning that the position of the laboratory operator and the cues around the maze remained unchanged throughout the test.

### Escape Acquisition (Days 1–5)

Each rat was tested in two independent trials per day for 5 days, each trial with a period of 90 s. Before each training, the rat was left on the platform for 15 s to be familiarized with the environment around the maze. Once the rat climbed the platform, they stayed there for 15 s. After that, it was removed from water, dried, and returned in its cage until the next trial. For each trial, latency to locate the hidden platform and the total swimming distances were measured representing spatial learning performance.

### Probe Trial (Day 6)

After 24 h of training, a probe trial was conducted on day 6 without the absence of the platform. During 90 s test, rat was placed into the water in the opposite quadrant and the number of target crossing and the time spent in the target quadrant were recorded by the tracking system to assess the spatial memory ability.

### Working Memory (Days 7–10)

After the probe trial test, the working memory evaluation was conducted. The procedure similar to the escape acquisition method was used, but the position of the hidden platform was changed daily. The starting position was randomly changed from trial to trial within a given day. Rats were submitted to two trials per day and were allowed to swim freely to search for the hidden platform for up to 90 s. The experimenters should guide the rat to stay on the platform for 15 s if it failed to find the platform. All rats were tested twice daily for 4 days, and the escape latency and the distance spent in the target quadrant were calculated for the evaluation of the working memory.

### Tissue Preparation

After final behavioral tests, rats were anesthetized and killed. The hippocampus tissues were collected rapidly and stored at −80°C until further assays.

### Biochemical Assays

In the hippocampus, AChE, ChAT, and SOD activities were measured using commercially available assay kits. Briefly, the hippocampus was homogenized in 10 × saline on ice, centrifuged (3,500 rpm/min) at 4°C for 15 min, and the supernatant was collected. AChE activity was determined using a thiol agent to form trinitrobenzene at 412 nm. ChAT activity was determined by using a commercial kit. All procedures completely complied with the manufacturer's instructions. The mixture was incubated at 37°C for 20 min and boiling water (100°C) was added to stop the reaction. It was centrifuged (4,000 r/m) for 10 min, and the suspension was used for color reaction. The SOD activity was based on its ability to inhibit the oxidation by superoxide anion free radical produced from the xanthine–xanthine oxidase system, measured at 560 nm.

### Statistical Analysis

All data analyses were carried out using SPSS (version 19.0). Values are expressed as mean ± standard error of the mean (SEM). Group differences in the escape latency and swimming distance during acquisition of Morris water maze test (MWM) trials were analyzed using repeated-measure two-way ANOVA. Other data were analyzed by one-way ANOVA and the least significant difference required after ANOVA. The value of *p* < 0.05 was considered to be significant.

## Results

### Body Weight

**Figure 3:**
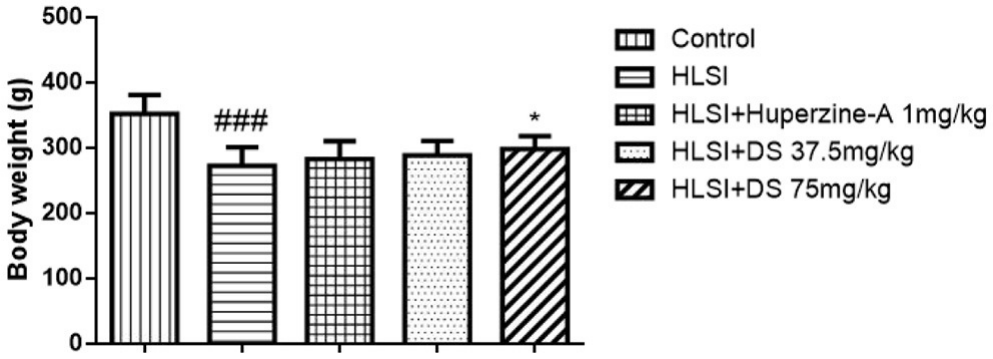
The effects of DS on the body weight after HLSI in rats. All values are means ± SEM (*n* = 8–10). The symbols represent: ^###^*p* < 0.0001 vs. the control group; *p < 0.05 vs. the HLSI group.

The body weight in the HLSI group decreased significantly (*p* < 0.001). However, after treatment with DS-1227 75 mg/kg, it was significantly reversed as compared with the control groups (Figure 3).

### Effect of DS on the Open-Field Test in Simulated Microgravity Induced by HLSI in Rats

**Figure 4:**
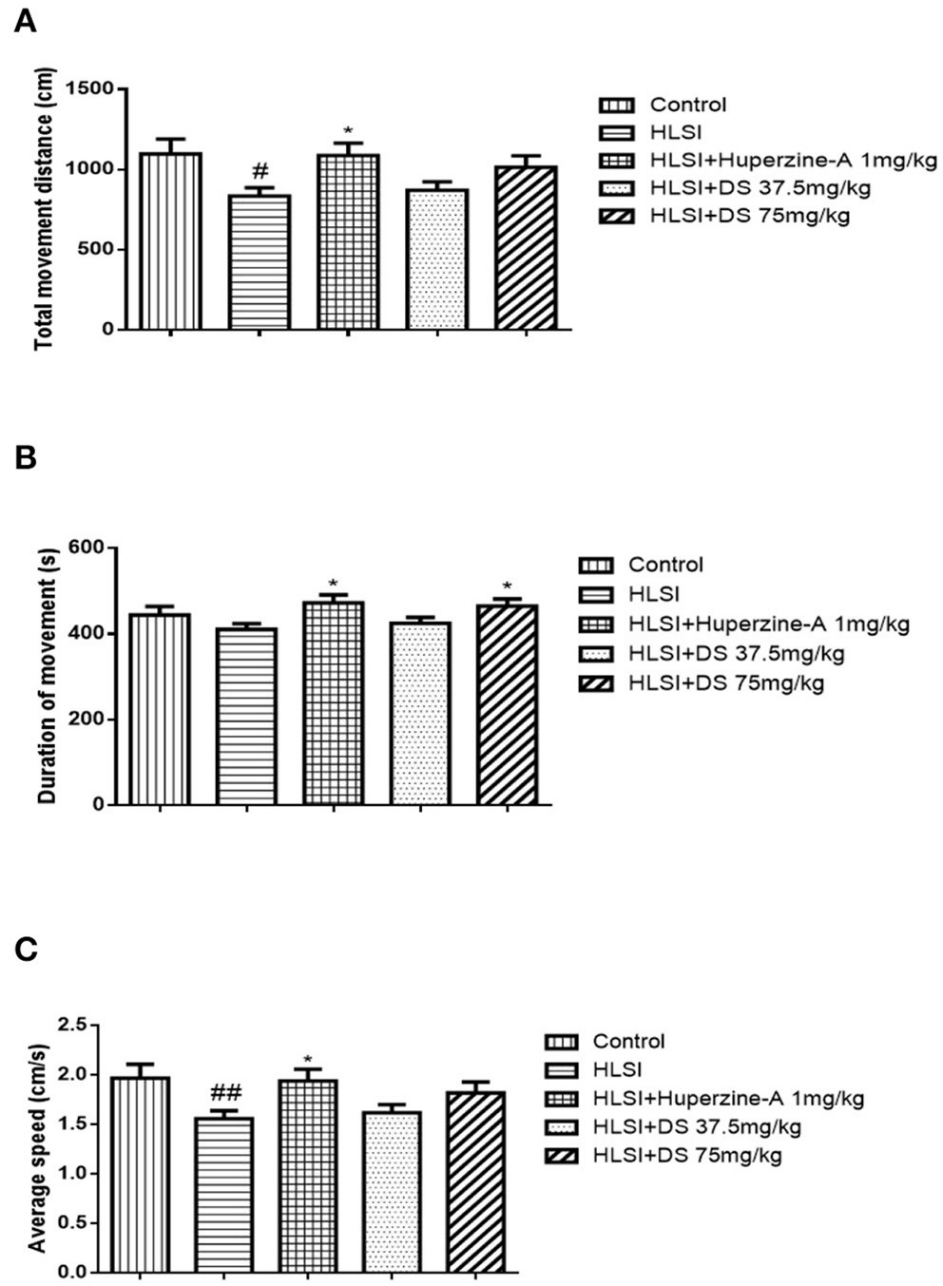
The effects of DS on the locomotor activity in the open-field test after HLSI in rats. The values are mean ± SEM (*n* = 8–9) for the following: **(A)** total movement distance, **(B)** duration of movement, and **(C)** average speed. The symbols represent: ^#^*p* < 0.05 and ^##^*p* < 0.01 as compared with the control group; *p < 0.05 as compared with the HLSI group.

**Figure 5:**
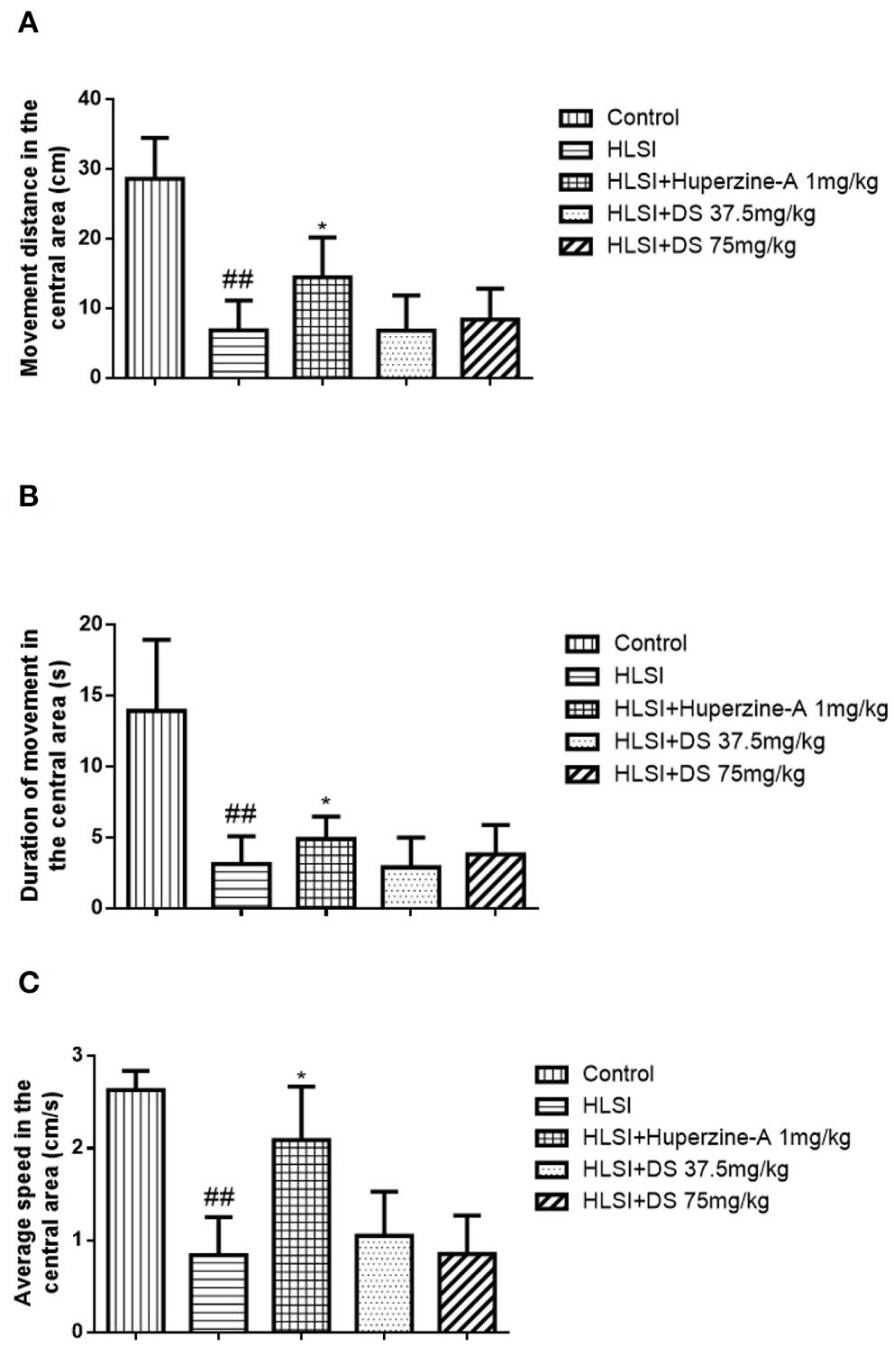
The effects of DS on the locomotor activity in the open-field test after HLSI in rats. The values are mean ± SEM (*n* = 8–9) for the following: **(A)** movement distance in the central area, **(B)** duration of movement in the central area, and **(C)** average speed in the central area. The symbols represent: ^##^*p* < 0.01 as compared with the control group; *p < 0.05 as compared with the HLSI group.

**Figure 6:**
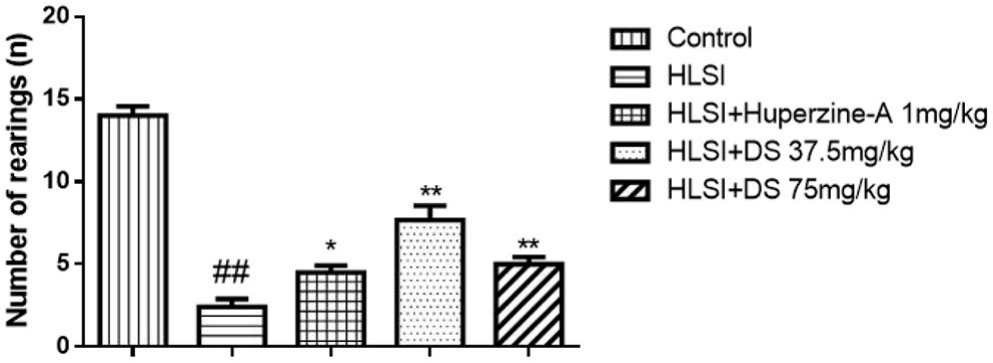
The effects of DS on the number of rearings in the open-field test after HLSI in rats. The values are mean ± SEM (*n* = 7). The symbols represent: ^##^*p* < 0.01 as compared with the control group; *p < 0.05 and **p < 0.01 as compared with the HLSI group.

In the open-field test (Figure 4), rats of the HLSI group covered less total movement distance, accompanied by less duration of movement and slower average speed compared with the control group (Figures 4A–C, *p* < 0.05 or *p* < 0.01). The DS treatment at 75 mg/kg reversed HLSI-induced decrease duration of movement. The huperzine-A demonstrated positive effects on HLSI-induced decrease in the total movement distance, less duration of movement, and slower average speed in rats (*p* < 0.05). In the center area of the open-field test, rats in the HLSI group covered shorter total movement distance, less duration of movement, and with slower average speed compared with the control group (*p* < 0.01) (Figures 5A–C). The huperzine-A treatment elicited positive effects by improving the locomotor activity in the HLSI rats in the center area (*p* < 0.05). Whereas, DS treatment showed a weak effect in enhancing the visit of the HLSI rats in the center area. The number of rearings in the HLSI group decreased significantly; however, DS or huperzine-A treatment increased the number of rearings in the open-field test, showing their protective effects on the HLSI rats (Figure 6; *p* < 0.05 or *p* < 0.01).

### Effect of DS on the Novel Object Recognition Test in Simulated Microgravity Induced by HLSI in Rats

**Figure 7:**
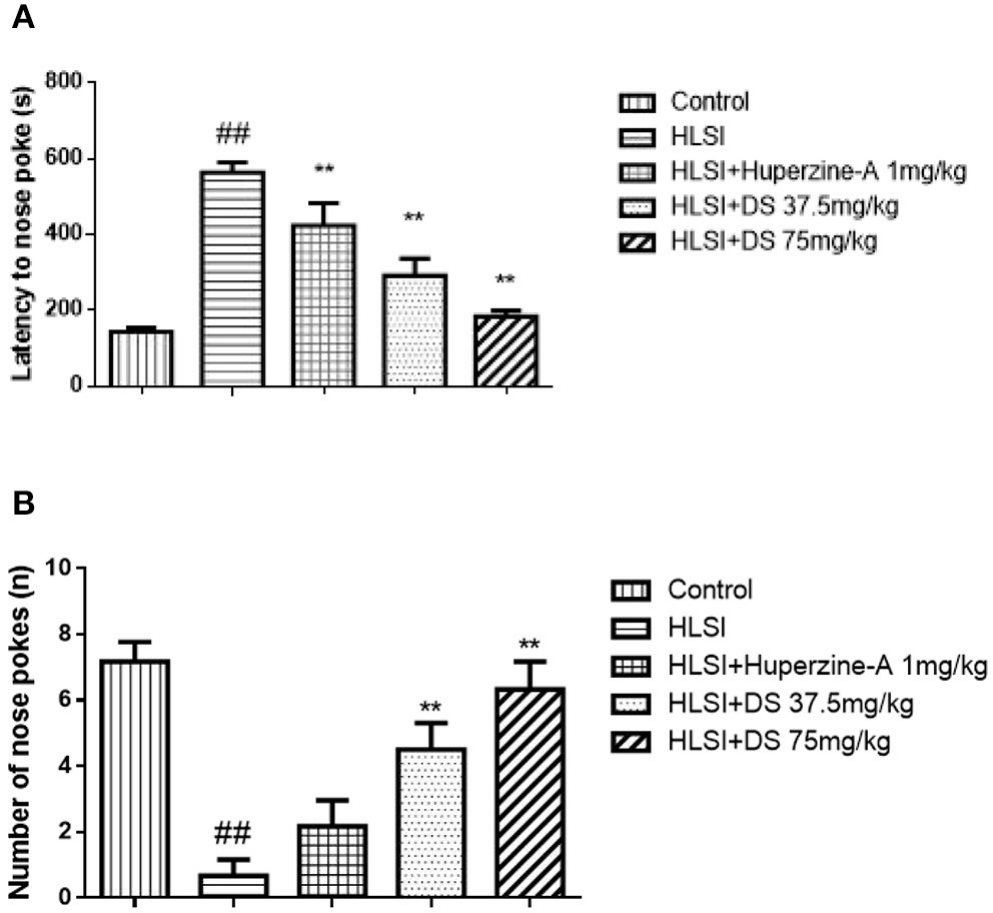
The effects of DS on the novel object recognition test after HLSI in rats. The values are mean ± SEM (*n* = 8–9) for the following: **(A)** latency of nose poke and **(B)** number of nose pokes. The symbols represent: ^##^*p* < 0.01 as compared with the control group; **p < 0.01 as compared with the HLSI group.

As presented in Figure 7, the latency to nose poke increased while it decreased in the HLSI rats compared with the control rats (*p* < 0.01). Both DS or huperzine-A treatment could significantly reversed the HLSI-induced damage to rats (*p* < 0.01).

### Effect of DS on the Forced Swimming Test in Simulated Microgravity Induced by HLSI in Rats

**Figure 8:**
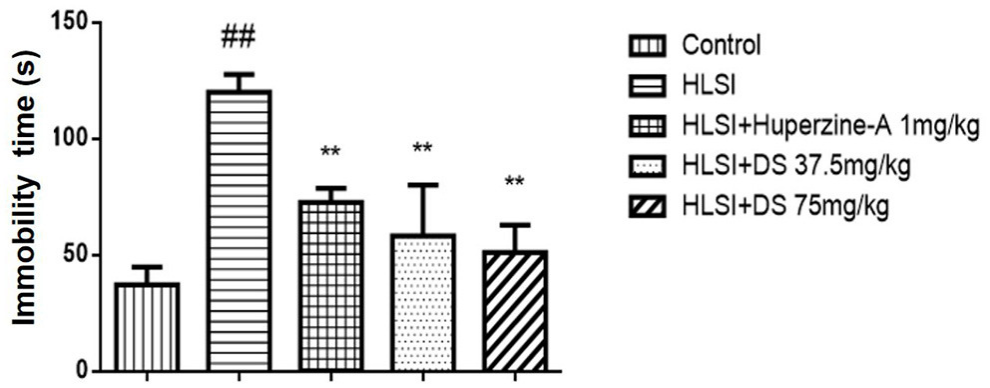
The effects of DS on the immobility time in the forced swimming test after HLSI in rats. The values are mean ± SEM (*n* = 8–9). The symbols represent: ^##^*p* < 0.01 as compared with the control group; **p < 0.01 as compared with the HLSI group.

The immobility time was increased in the HLSI rats as compared with the control rats (*p* < 0.01); however, DS or huperzine-A treatment significantly reversed the HLSI-induced despair in the force swimming test in rats (*p* < 0.01, Figure 8).

### Effect of DS on Spatial Learning and Memory in the MWM Test in Simulated Microgravity Induced by HLSI in Rats

**Figure 9:**
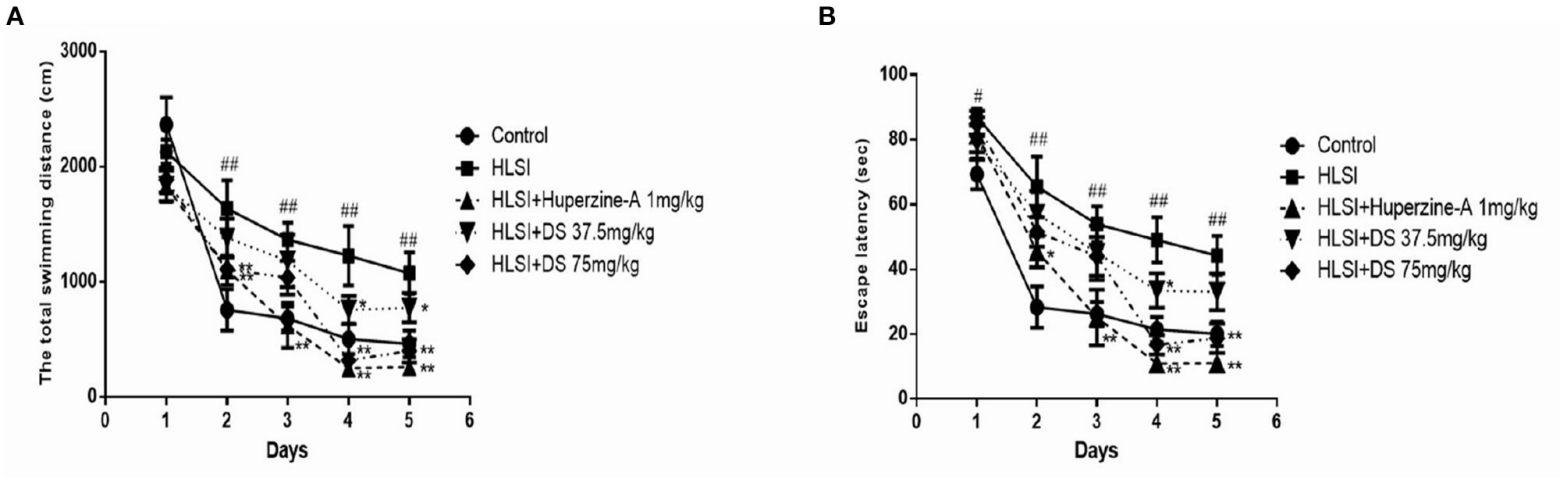
The effects of DS on the spatial memory in the MWM test after HLSI in rats. All values are mean ± SEM (*n* = 8–10) for the following: **(A)** total swimming distance and **(B)** escape latency. The symbols represent: ^#^*p* < 0.05 and ^##^*p* < 0.01 vs. the control group; *p < 0.05 and **p < 0.01 vs. the HLSI group.

In the MWM test, our results revealed significant differences on the swimming distance and escape latency between training days and among groups. The rats in the HLSI group showed significantly higher values regarding the total swimming distance and the escape latency as compared with the control group from days 3 to 5 (Figures 9A,B). Administration of DS (37.5 and 75 mg/kg) in rats reversed the HLSI-induced increase in the total swimming distance and the escape latency in the MWM test (*p* < 0.05 or *p* < 0.01). Whereas, administration of huperzine-A (1 mg/kg) in rats induced a significant reduction in the total swimming distance and the escape latency as compared with the HLSI group (*p* < 0.05).

**Figure 10:**
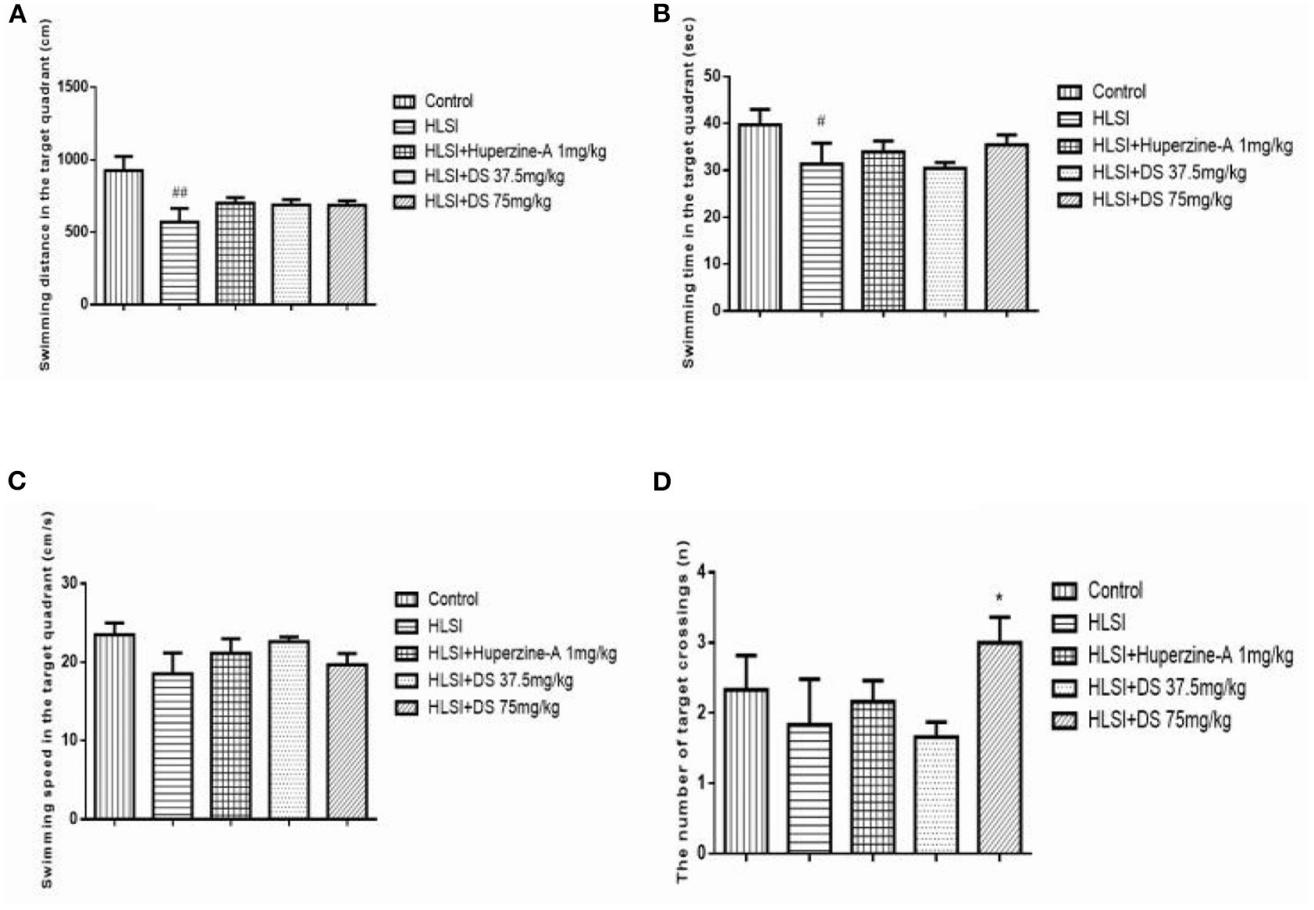
The effect of DS on memory of rats in probe trial of the MWM test. All values are means ± SEM (*n* = 7–8). In the target quadrant, parameters noted were as follows: **(A)** swimming time, **(B)** swimming distance, **(C)** swimming speed, and **(D)** the number of target crossings. The symbols represent: ^#^*p* < 0.05 and ^##^*p* < 0.01 vs. the control group; *p < 0.05 vs. the HLSI group.

In the probe trial, rats of the HLSI group spent less time and swam the least distances in target quadrant (*p* < 0.05) (Figures 10A,B). The DS showed non-significant reversal of this decline. Likewise, non-significant differences in speed and number of target crossings between control and HLSI rats were evident (Figures 10C,D). However, the rats after treatment with DS (75 mg/kg) demonstrated a significant rise in the number of target crossing as compared with the HLSI group (*p* < 0.05). Huperzine-A had no effect on either the swimming speed or the number of target crossing in the target quadrant in the probe test.

**Figure 11:**
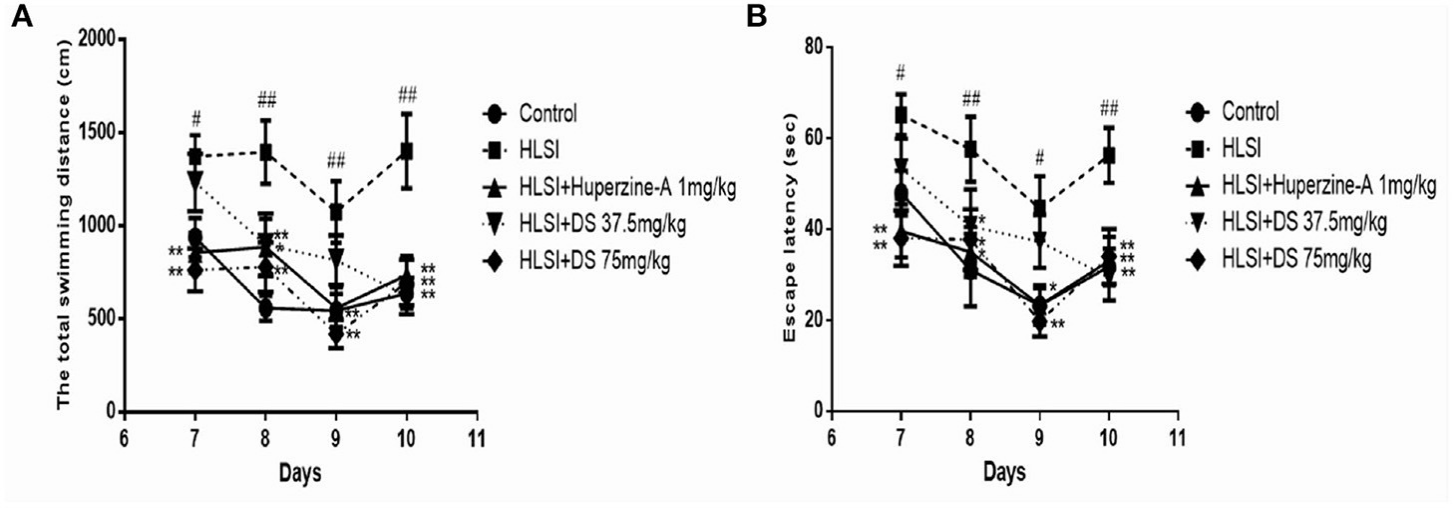
The effect of DS on the working memory of rats in the MWM test. All values are means ± SEM (*n* = 8–9) for the following: **(A)** the total swimming distance, **(B)** the escape latency. The symbols represent: ^#^*p* < 0.05 and ^##^*p* < 0.01 vs. the control group; *p < 0.05 and **p < 0.01 vs. the HLSI group.

In the MWM test during 7–10 days, all rats were subjected to the working memory test, to assess working or trial-dependent learning and memory (Figures 11A,B). On day 7, DS (75 mg/kg) revealed a shorter distance and time to find the platform in novel position (*p* < 0.01). Likewise, on days 8–10, the latency still showed a shorter total swimming distance and escape latency than that of the HLSI group (day 8, *p* < 0.01; day 9, *p* < 0.01; day 10, *p* < 0.01). However, the total swimming distance and escape latency of the HLSI rats exhibited a tendency toward an increase during the MWM test.

### Effect of DS on Acetylcholine-Related Enzymes in Simulated Microgravity Induced by HLSI in Rats

**Figure 12:**
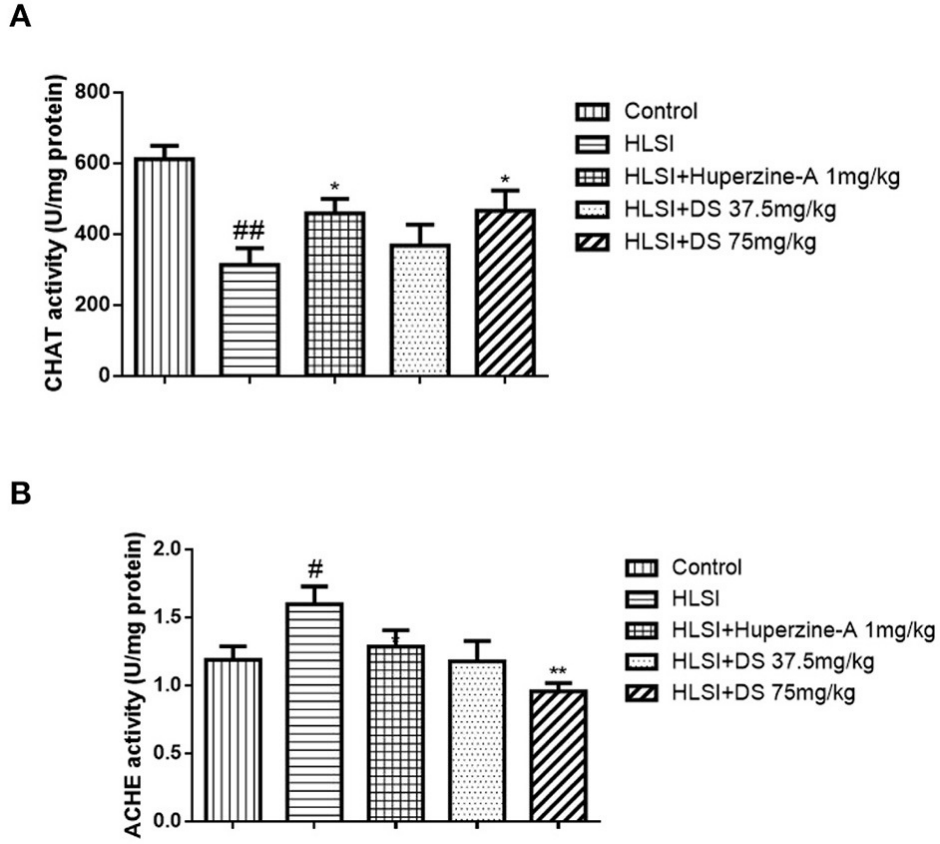
The effect of DS on acetylcholine-related enzymes in the hippocampus of rats. All values are means ± SEM (*n* = 6–8) for the following: **(A)** the AChE activity in the hippocampus and **(B)** the ChAT activity. The symbols represent: ^#^*p* < 0.05 and ^##^*p* < 0.01 vs. the control group; *p < 0.05 and **p < 0.01 vs. the HLSI group.

Figures 12A,B depicts the effect of DS on acetylcholine-related enzyme changes on the HLSI-induced cognitive deficit. Results showed that in rat hippocampus, AChE activity (*p* < 0.05) was significantly higher whereas ChAT activity (*p* < 0.01) decreased in the HLSI group compared with the control. After treatment with DS (75 mg/kg) for 2 weeks, AChE activity (*p* < 0.01) was reduced whereas the ChAT activity (*p* < 0.05) elevated significantly (*p* < 0.05 or *p* < 0.01). Huperzine-A (1 mg/kg) also produced an increase in ChAT activity (*p* < 0.05).

### Effect of DS on SOD Activity in Simulated Microgravity Induced by HLSI in Rats

**Figure 13:**
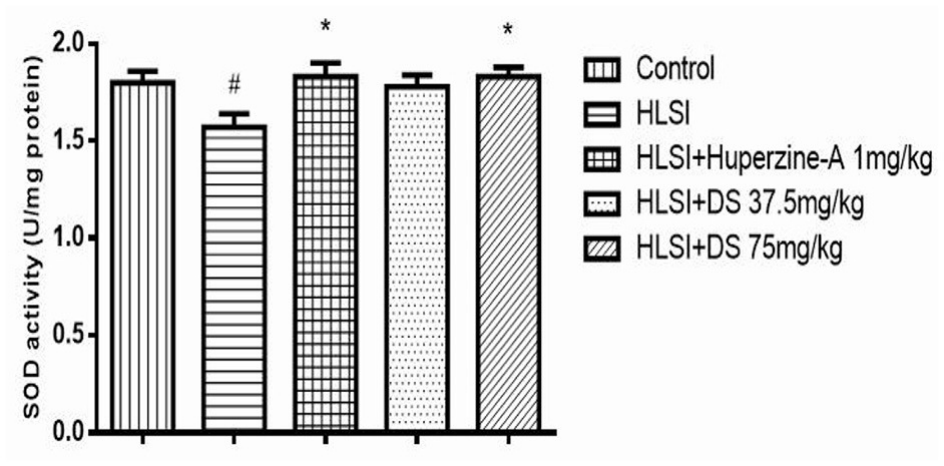
The effect of DS on SOD activity in the hippocampus of rats. All values are means ± SEM (*n* = 6). The symbols represent: ^#^*p* < 0.05 vs. the control group; *p < 0.05 vs. the HLSI group.

As verified in Figure 13, SOD activity in the hippocampus of the HLSI group was significantly lower than the control group (*p* < 0.05). After treatment with DS (75 mg/kg), SOD activity was significantly increased in rats submitted to HLSI insult, up to levels similar to the control (*p* < 0.05). The SOD activity of the huperzine-A group also increased significantly (*p* < 0.05).

## Discussion

In our study, protective effects of DS were evident in depressive-like behavior and cognitive dysfunction in rats after 14 days to HLSI exposure. Moreover, the protective effect of DS was highly significant at higher dose.

The DS is acquired from *Panax ginseng* C. A. Mey and predominantly contains PPT and PPD. Earlier studies demonstrated that they possesses various pharmacological activities, including anti-inflammatory, antitumor, and neuroprotective effects (17). Our research also showed that ginsenoside Rg1 and Rb1, PPD, and PPT were able to ameliorate memory impairments in mice induced by scopolamine (18) and chronic sleep deprivation (8). Ginsenoside Rg1 significantly reversed the low Bcl-2 with concomitant increase in Bax expression in the prefrontal cortex (PFC) in rats induced by chronic restraint stress (13), indicating its biological effect on anti-oxidation. Wu Xiaorui et al. showed that DS improved the cognition dysfunction in rats after 42 days of simulated long-duration spaceflight environment exposure (19). Thus, in the present study, we aimed to explore the beneficial effects of DS on depressive-like behaviors and memory dysfunction in rats after 14 days of simulated microgravity.

HLS in animals provides a popular animal model to simulate microgravity on earth. Rats exposed to HLS for 2 weeks showed a significantly lower performance in the water maze test, and Nissls and Golgi-Cox staining showed significant changes in hippocampus CA1 neuronal cytomorphometry (20). Our previous studies also strongly supported poor performance of rats in the Morris water maze and shuttle box test after encountering HLS for 2 weeks (21, 22). Simultaneously, isolation, as a kind of psychosocial stress (23), has a serious influence on emotion and cognition function. Gaskin et al. showed that 40 days of post-weaning social isolation caused novel object recognition impairment in isolation-reared rats compared with the group-housed rats (24). Likewise, in our study, HLSI-induced significant damage in novel object recognition test. Thus, a combination of hindlimb suspension and isolation provides an appropriate method to simulate the extreme environment of space.

It is known that simulated weightlessness can cause depressive-like behavior in rats (25). The simulated weightlessness led to weight loss, decreased locomotor activity, prolonged latency to nose poke, decreased number of nose poke, and increased immobility time in rats, indicating that the rats have depressive-like symptoms. The administration of DS (75 mg/kg) improved the weight loss caused by simulated weightlessness in rats. The open-field test, mainly used to study the exploratory behavior of animals and to test the ability of animals to perform in a relatively open and unfamiliar environment (26). Rodents suffering from depression, due to avoidance and fear of unfamiliar environment, mainly reduce their activities in the central area of the open field and increase their peripheral activities (27). In the open-field test, DS (37.5, 75 mg/kg) antagonized the depressive-like behavior caused by simulated weightlessness, as reflected by increasing duration of movement, and number of rearing, of which 75 mg/kg dose of DS was more effective. Huperzine-A also improved the locomotor ability of rats. The novel object recognition test is suitable to study the behavior of animals exploring novel things in a familiar environment, and its latency of nose poke reflects the exploration ability of rodents (15). The present experiment showed that HLSI can reduced the ability of mice to explore new things, while DS 37.5 and 75 mg/kg and huperzine-A can reduce the latency of nose poke thereby increasing the number of nose pokes. The effect of DS 75 mg/kg appeared to be better than its lower dose. The forced swimming test based on animals escaping from the water and struggle constantly. Depressed animals will show less struggling or even giving up struggling activity (28). In FST, DS (37.5, 75 mg/kg) and huperzine-A also reversed the increase in immobility time induced by HLSI. These behavioral experiments have indicated that DS possesses appreciable antidepressant effect.

The MWM is designed to test spatial learning and working memory and is a widely accepted method for assessing the therapeutic potential of new drugs for cognitive dysfunction (29). When the test is repeated several times, the changes in latency and distance covered to reach the platform are suitable indicators for the learning and memory abilities of the animals (29, 30). In our study, the HLSI rats demonstrated longer latency and swimming distance in the escape acquisition test. Moreover, the number in target crossings, the time, and the swimming distance toward the target quadrant were decreased in the HLSI group in the probe trail test, meaning that HLSI for 2 weeks possibly impairs spatial memory. After treatment with DS, the performance over the two trials of rats in MWM was significantly improved. It is very interesting to note that the HLSI rats required longer time to find the platform as compared with the control rats in the working memory task in the MWM test. The term working memory, defined as the capacity to maintain a limited amount of information through active rehearsal (1), provides temporary storage and manipulation of the information (31). It is well-established that working memory is crucial for arithmetic problem solving (32), language learning (33), and long-term memory consolidation (34). Moreover, spatial working memory is necessary for actions to guide thought processes. Previous research showed that participants' accuracy and reaction time decreased significantly in head-down bed rest, especially the Thurston's card rotation and cube mental rotation tasks, which usually measure the working memory. Our study showed for the first time that DS could improve spatial learning and working memory impairment induced by HLSI.

Indeed, cholinergic nervous system plays a vital role in the learning and memory processes. Both ChAT and AChE are the most specific indicators for monitoring the functional state of cholinergic neurons in the central nervous system. Reduced ChAT activity and increased AChE activity have been reported in the brains of patients with AD (35). Protecting central cholinergic neurons from functional degenerative disorders and maintaining the activities of ChAT and AChE in the neurons may be valuable for the prevention of the development or progression of AD and/or other chronic brain degenerative diseases (36). Thus, the activities of ChAT and AChE are popularly used to appraise the cognitive function in animals. Our results showed that HLSI did affect these two enzymes, especially ChAT activity which was reduced in the hippocampus of the HLSI group. The DS ameliorated the increased AChE activity and increased ChAT activity in the hippocampus. These results suggest that cognitive damage caused by HLSI is closely associated with dysfunction of the cholinergic system. In the brain of AD patients, changes in free radical-induced damages and SOD activity or expression have been reported (37). Simulated microgravity induced an oxidative imbalance in astronauts and other animal models. Our study confirmed that SOD activity was significantly decreased in the hippocampus of rats submitted to HLSI insult, indicating the activation of the oxidative stress system after exposure to microgravity.

Based on our findings, HLSI significantly induced the loss of locomotor rearings and exploration to novel object, despaired to survival in forced swimming, impaired reference, and working memory. Intragastric administration of DS could improve these depressive-like behaviors and cognitive impairments. The mechanisms were associated with inhibition of AChE activity and activation of ChAT and SOD activities in the hippocampus of rats. DS might be a potential agent for preventing and treating depressive-like behaviors and memory loss, especially for the deficiency of working memory under spaceflight.

## Conclusions

In summary, our study provides evidence that DS from *Panax ginseng* significantly alleviated the depressive-like behaviors and cognitive impairment induced by HLSI in rats, and the psychotic and neuroprotective effects may be related to mediate the cholinergic system and oxidative stress, including activation of ChAT and SOD activities and inhibition of AChE activity. Further investigations on the protective activity of DS in depressive-like behaviors and cognitive impairments induced by weightlessness are needed to illustrate deep mechanisms and may provide a useful candidate during spaceflight.

## Data Availability Statement

The original contributions presented in the study are included in the article/Supplementary Material, further inquiries can be directed to the corresponding author/s.

## Ethics Statement

The animal study was reviewed and approved by The committee for the Care and Use of Laboratory Animals of IMPLAD, CAMS & PUMC, China (No. 2016515).

## Author Contributions

QW designed the project and wrote this manuscript. MW and YC helped in writing the manuscript. LD, SL, and YZ performed the experiments. SC, SY, and XL helped in conducting the research. WH and HZ helped in analyzing the data and contributed to the discussion. AP helped in editing the manuscript. All authors contributed to the article and approved the submitted version.

## Conflict of Interest

The authors declare that the research was conducted in the absence of any commercial or financial relationships that could be construed as a potential conflict of interest.
