# Supplementary figures and images for: Dammarane Sapogenins Improving Simulated Weightlessness-Induced Depressive-Like Behaviors and Cognitive Dysfunction in Rats
Source: Front Psychiatry. 2021 Mar 26;12:638328. doi: 10.3389/fpsyt.2021.638328 (PMC8032884; doi:10.3389/fpsyt.2021.638328)

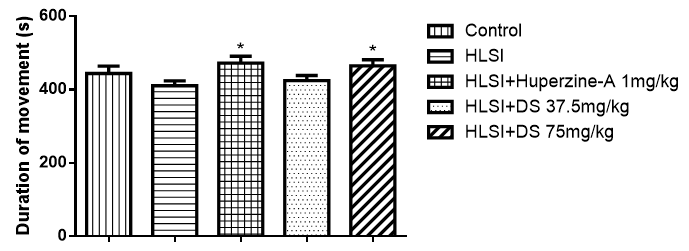

Supplement: Supplementary file 1 [file Image_1.TIF]

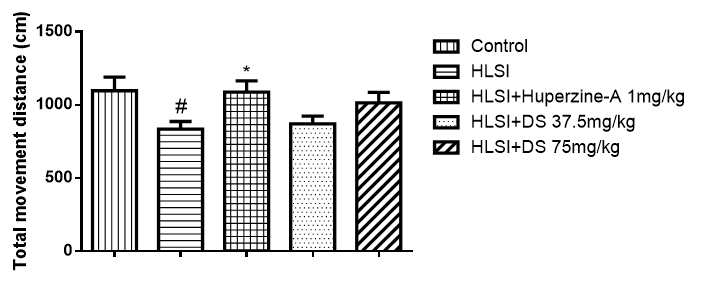

Supplement: Supplementary file 2 [file Image_2.TIF]

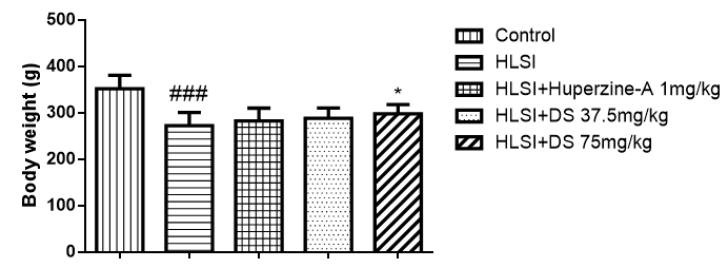

Supplement: Supplementary file 3 [file Image_3.TIF]

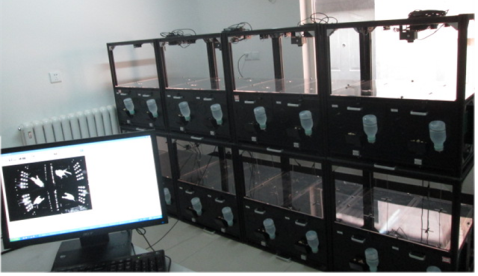

Supplement: Supplementary file 4 [file Image_4.TIF]

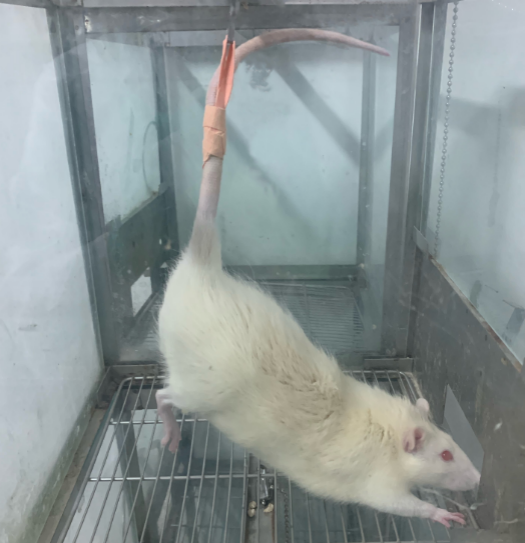

Supplement: Supplementary file 5 [file Image_5.TIF]

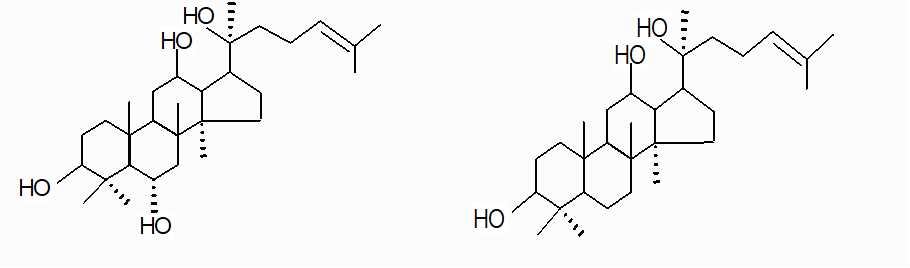

Supplement: Supplementary file 6 [file Image_6.TIF]
